# Pharmacogenetic research in Kazakhstan

**DOI:** 10.5195/cajgh.2013.87

**Published:** 2014-01-24

**Authors:** Elena Zholdybayeva, Aisha Iskakova, Aliya Romanova, Erlan Ramanculov, Kuvat Momynaliev

**Affiliations:** 1 National Center for Biotechnology, Astana, Kazakhstan; 2 Nazarbayev University, Astana, Kazakhstan

**Keywords:** pharmogenomic research, genotyping, allelic frequencies, Kazakhstan

## Abstract

**Introduction:**

Pharmacogenomics is an emerging field of medicine that combines genetics and pharmacology. Pharmacogenomic research is relatively new in Kazahkstan, but, in recent years, significant progress has been made in this field. The National Scientific Laboratory for Biotechnology has launched several government-funded research projects focused on finding genetic markers that determine susceptibility to various drugs. Another goal of pharmacogenetic research in the laboratory is to find the pharmacogenomic markers that target cardiovascular diseases, accounting for allelic frequencies in selected genes in the Kazakh population. In addition, pharmacogenomic testing kits allow patients to choose the drug dosage. For example, the drug Warfarin has been developed within the framework of the “Technology Commercialization Project,” funded jointly by the Ministry of Education and Science of the Republic of Kazakhstan and the World Bank.

**Material and methods:**

The pharmacogenomic studies were conducted using the real-time PCR and direct DNA sequencing. DNA was isolated from venous blood or buccal cells, collected from patients.

**Results:**

To date, we have identified the most promising areas of research in the field of pharmacogenomics in Kazakhstan. The allelic frequencies of a number of polymorphisms in the Kazakh population have been calculated (CYP2C9, CYP2C19, CYP3A4, VKORC1, CYP4F2, GGCX, CYP2D6, CYP1A2, NAT2, GSTP1, SLC47A1). A unique repository of DNA samples was established and is being replenished during the implementation of aforementioned projects. Development of the testing kit for individual selection of Warfarin dosage is nearing completion. A patent, named “Method of Selection Based Dose Warfarin Genotyping for the Kazakh Population” has been recently obtained. An application for another patent, titled “Express Method of Correction of Warfarin Dosing, Based on Real-time PCR” has received positive evaluation. The results of domestic pharmacogenomic studies will allow a more rational selection of drugs and their dosage regimens specific to the Kazakh population.

